# An fMRI Study of Concreteness Effects during Spoken Word Recognition in Aging. Preservation or Attenuation?

**DOI:** 10.3389/fnagi.2015.00240

**Published:** 2016-01-12

**Authors:** Tracy Roxbury, Katie McMahon, Alan Coulthard, David A. Copland

**Affiliations:** ^1^University of Queensland Centre for Clinical Research, University of QueenslandBrisbane, QLD, Australia; ^2^School of Health and Rehabilitation Sciences, University of QueenslandBrisbane, QLD, Australia; ^3^Centre for Advanced Imaging, University of QueenslandBrisbane, QLD, Australia; ^4^Department of Medical Imaging, Royal Brisbane and Women's HospitalBrisbane, QLD, Australia; ^5^Academic Discipline of Medical Imaging, University of QueenslandBrisbane, QLD, Australia

**Keywords:** aging, concreteness, fMRI, concrete, abstract, spoken word, auditory

## Abstract

It is unclear whether healthy aging influences concreteness effects (i.e., the processing advantage seen for concrete over abstract words) and its associated neural mechanisms. We conducted an fMRI study on young and older healthy adults performing auditory lexical decisions on concrete vs. abstract words. We found that spoken comprehension of concrete and abstract words appears relatively preserved for healthy older individuals, including the concreteness effect. This preserved performance was supported by altered activity in left hemisphere regions including the inferior and middle frontal gyri, angular gyrus, and fusiform gyrus. This pattern is consistent with age-related compensatory mechanisms supporting spoken word processing.

## Introduction

Word retrieval deficits are well documented in healthy older individuals (Burke and Shafto, 2004, 2008) while word knowledge (Verhaeghen, 2003) and language comprehension abilities appear less susceptible to aging effects (Tyler et al., 2010). The present study is focused on how older adults process concrete vs. abstract words, where there are conflicting findings of (1) a reduction in concreteness effects (the processing advantage observed with concrete over abstract words) in recall and recollection memory tasks (Rissenberg and Glanzer, 1987; Peters and Daum, 2008) vs. (2) a preservation of concreteness effects with lexical tasks (Huang et al., 2012). Given age-related changes in brain structure and function it is also not clear whether recruitment of different neural mechanisms is necessary during concrete vs. abstract word processing in order to ensure preserved functionality in older adults.

There is an extensive literature demonstrating that concrete words are more easily recalled in memory tasks (Paivio, 1971) and tend to be responded to more quickly and accurately than abstract words during lexical decision tasks (James, 1975; Kroll and Merves, 1986). Historically, concreteness effects and conceptual differences between concrete and abstract words have been investigated and explained by two prominent theories of cognition: dual coding theory (Paivio, 1971, 1986) and context availability theory (Schwanenflugel and Shoben, 1983). Dual coding theory proposes that the processing advantage for concrete words is due to the dual system representation (verbal and nonverbal) available to concrete but not abstract words (Paivio, 1971, 1986) while context availability attributes the processing efficiency observed with concrete words to an increased amount of available context, associated with concrete but not abstract words due to their richer set of semantic-based representations (Schwanenflugel and Shoben, 1983). However, the results from neuroimaging studies investigating concrete and abstract processing have been inconsistent and a definitive interpretation in support of either of these two dominant theories has thus far proved elusive.

In a meta-analysis of brain imaging studies involving concrete (perceptual) and abstract (verbal) conceptual processing, Binder et al. (2009) found that concrete words were strongly associated with a wide network of regions including the left angular gyrus (AG), the left fusiform (occipitotemporal) gyrus, left superior frontal gyrus (SFG), left middle frontal gyrus (MFG), left posterior cingulate, and right AG. Abstract words were associated with left anterior superior temporal sulcus (aSTS) and left inferior frontal gyrus (IFG) (Binder et al., 2009). A meta-analysis by Wang et al. (2010) reached similar conclusions with concrete words eliciting left hemisphere activity in the posterior cingulate, fusiform, precuneus, and parahippocampal regions. Abstract word processing also elicited left hemisphere activity but this was in the left IFG, middle temporal gyrus (MTG), and superior temporal gyrus (STG) (Wang et al., 2010). Findings from imaging studies have been variously used to support dual coding theory (Binder et al., 2005; Sabsevitz et al., 2005), or dual coding and context availability theory (Fiebach and Friederici, 2004). Yet other studies showed no differences in activity for concrete compared to abstract words (Kiehl et al., 1999; Perani et al., 1999; Grossman et al., 2002; Noppeney and Price, 2004), which makes an interpretation in support of either theory problematic. Pexman et al. (2007) investigated concrete and abstract processing during a semantic categorization task and evaluated their findings within the frameworks of dual coding and context availability theory. Their results showed increased widespread activity for abstract words compared to concrete words and this was observed in brain regions normally associated with semantic-based conceptual representations including temporoparietal and frontal cortex. Since neither dual coding nor context availability theory predict increased activity for abstract words, Pexman et al. (2007) suggested that their findings were instead more compatible with Barsalou's perceptual symbol systems (Barsalou, 1999). In this strong embodiment theory of semantic representations, abstract words are expected to activate similar semantic-based regions to concrete words but with differences in the focus of the situational content. Thus, concrete words will have a more focused referent while the situational focus for abstract concepts is more distributed due to a more complicated set of referents (Barsalou, 1999; Barsalou and Wiemer-Hastings, 2005).

A modified theory of embodiment, termed *embodied abstraction* is a recent view put forward by Binder and Desai (2011) which proposes that conceptual information is represented in multiple levels in sensory, motor and affective systems and undergoes a process of abstraction from these inputs. They suggest that conceptual information develops in modality-specific representations (located near sensory, motor, and emotional networks) and interacts with modality-independent (supramodal) systems, located in the temporal and inferio-parietal lobes. These higher-level cortical regions serve to bind representations from different modalities with various levels of access activated in response to determining variables such as context, familiarity, or task demands (Binder and Desai, 2011). According to this view, the comprehension of conceptual information undergoes a process of gradual abstraction such that concrete words, which are associated more with sensory-motor experiential information will require less detailed simulations than abstract words. Other related theories have argued that processing abstract words is associated with greater emotional processing and associated neural mechanisms (Vigliocco et al., 2014).

Results from behavioral studies investigating concreteness and aging have provided mixed results with some reporting increases in the effects of concreteness with aging (Rowe and Schnore, 1971; Witte and Freund, 1976) while others have shown an attenuation of the effect (Rissenberg and Glanzer, 1987), possibly attributable to reduced cognitive function in underlying processes, such as memory. A cross-sectional study across three age groups (mean ages 21, 42, and 61 years) investigated concreteness effects and verbal memory (Peters and Daum, 2008). Concrete words were better recollected than abstract words in the three age groups, supporting the expected concreteness effect. However, they observed an attenuation of the effect in the older age group. While recollection of concrete words showed a steady, continuous decline with age, a reduction in the recollection of abstract words only occurred from the young to the middle-aged group. No further reduction was observed for the older group resulting in the attenuated concreteness effects (Peters and Daum, 2008).

To date, there have been very few neuroimaging studies which have directly investigated concrete and abstract processing effects in aging. Processing differences between young and older adults when making semantic judgments on concrete and abstract words, were investigated in an fMRI study conducted by Stebbins et al. (2002). The findings of this study showed hemispheric differences between the two groups with the young adults eliciting greater activity in the left hemisphere compared to the older adults. In a PET study, Whatmough et al. (2004) also investigated semantic judgments for concrete and abstract words in a group of older adults. They found that left lateral temporal cortex was similarly activated for both word types when compared to a baseline condition. However, a direct comparison between concrete and abstract conditions showed that concrete words elicited activity in left fusiform while for abstract words, the activity was observed in right fusiform regions. However, as Whatmough et al. (2004) did not include a comparison group of young adults, it is unclear whether their results reflected age-related neural differences.

Shafto et al. (2012) employed an auditory lexical decision task in a group of healthy young and older adults to investigate language comprehension function and specifically factors of (i) semantics (as measured by imageability) and phonology (as measured by phonological competition) in aging (Shafto et al., 2012). While both groups responded more quickly to words with high imageability, only the older adults showed an increased behavioral sensitivity to low imageability words and this was associated with greater activity in the left MTG. The findings of Shafto et al. (2012) suggest that preserved processing of abstract vs. concrete words in older adults may be associated with age-related compensatory brain activity but the balance between discrete processing components may change, such that an age-related upregulation for the semantic task serves a compensatory role while the differences that occur in response to phonological processes may not (Shafto et al., 2012 but see Geva et al., 2012). Meinzer et al. (2012) observed a difference in brain activity between young and older adults for semantic but not phonemic fluency, with older adults showing an upregulation of the right IFG and a reduced behavioral performance compared to the young adults, further suggesting that age-related changes in language processing may vary as a function of semantic vs. phonological processing. Meinzer et al. (2012) interpret this finding as reflecting ineffective compensation for left IFG semantic functions. While recruitment of prefrontal structures appears to play a role in age-related changes in semantic processing, other regions also appear to be integral in maintaining preserved language functions and should not be overlooked (Shafto et al., 2012) and it remains unclear as to whether age-related differences that may be associated with the processing of concrete vs. abstract words reflect compensatory or inefficient mechanisms.

Conceptual theories of aging and cognitive performance such as dedifferentiation (Reinert, 1970; Baltes et al., 1980; Lindenberger and Baltes, 1994) and compensation (Reuter-Lorenz and Park, 2010) have been used to explain functional differences between the brain activity elicited by young compared to older adults when preforming the same task (Cabeza et al., 2002; Wingfield and Grossman, 2006; Shafto and Tyler, 2014). The dedifferentiation hypothesis proposes that increased, widespread activity in healthy older brains is necessary and occurs as distinct cortical regions lose their specialized functionality (Li and Lindenberger, 1999). The compensation hypothesis meanwhile proposes that increased activity of additional brain regions serves as a strategic mechanism (Cabeza et al., 1997, 2002) to enable a preserved functional performance while compensating for neurocognitive decline in the aging brain (Park et al., 2003, 2004; Reuter-Lorenz and Park, 2010).

In the present study, we employed an fMRI task to investigate the spoken word comprehension of concrete and abstract words in young and older adults performing a lexical decision task. We wanted to test competing hypotheses: that concreteness effects are attenuated with age (as per Peters and Daum, 2008) vs. the preservation of the effect consistent with the findings of Huang et al. (2012). We also wanted to determine whether a preserved performance by the older adults was associated with age-related differences in neural activity between groups. In view of structural changes in prefrontal regions associated with aging, we predicted that a preserved performance by older adults would be accompanied by changes in the underlying substrates associated with concrete and abstract processing in healthy young adults and reported previously in Roxbury et al. (2014). We employed an auditory lexical decision task with a novel pseudoword condition to investigate the different processing mechanisms associated with concrete and abstract words in young and older adults considering the key brain regions previously reported as being reliably associated with concrete and abstract processing (Binder et al., 2009; Wang et al., 2010). Given the evidence for upregulation of the contralateral prefrontal cortex (PFC) by healthy older adults during semantic fluency tasks (Meinzer et al., 2009, 2012), we also examined age-related differences in the right IFG.

## Methods

### Materials

A total of 120 polysyllabic words and pseudowords were included in the stimuli list. Sixty of the 120 were pseudowords while the other 60 were English real words. The real word condition was manipulated to include 30 high imageability, concrete nouns (e.g., wallet, hospital) and 30 low imageability, abstract nouns (e.g., saga, rarity). Real word stimuli were controlled for a number of variables including (i) spoken word frequency, (ii) written word frequency, (iii) phoneme length, (iv) phonological neighborhood density, (v) concreteness, (vi) imageability, and (vii) number of syllables. There was no statistical significance difference (*p* > 0.05) between abstract and concrete words for any of the variables except concreteness and imageability (for stimuli characteristics and statistical significance, refer to Table 1 in Roxbury et al., 2014). All pseudoword stimuli conformed to English phonological rules. Mean average durations were calculated for the three conditions [concrete, 724 ms (SD 105 ms); abstract, 772 ms (SD 119 ms) and pseudowords, 780 ms (SD 93 ms)] and a Kruskal-Wallis test (Kruskal and Wallis, 1952) revealed no statistical, significant difference (*p* > 0.05).

The 60 pseudowords were matched for number of phonemes and syllables with the 60 real words, and syllable boundaries determined from the MRC database (Wilson, 1988). In order to control for syllable frequency, the syllables from each real word were re-combined to make the pseudowords whilst ensuring that their original constituent position remained constant (Valdois et al., 2006). As a result, 60, phonologically legal, opaque polysyllabic pseudowords were created as per Raettig and Kotz (2008), resulting in 120 items as detailed in Roxbury et al. (2014). A native female English speaker recorded all stimuli digitally in a soundproofed room using a Rode NTK condenser.

### Participants

Twenty-six healthy young and 24 healthy older adults were initially recruited to the study. The young participants have previously been reported in Roxbury et al. (2014). Recruitment for the older participants was achieved through advertising flyers in local community centers and in the University of Queensland newsletters. Written consent was obtained from all participants with each receiving $30 in reimbursement. Following the scanning session, all scans were reviewed by a neuroradiologist for possible structural abnormalities. Incidental findings of clinical significance were noted in six scans (three younger adults and three older adults) and these subjects excluded. A further six young adults and four older adults were also excluded from analysis due to technical and subject compliance issues. As a result, 17 young adults aged between 18 and 35 years (*M* = 27.35, *SD* = 5.1; 8 males), and 17 older adults ranging from 64 to 83 years (*M* = 71, *SD* = 5.07; 6 males) were included in the present study.

All participants were right hand dominant according to the Edinburgh Handedness Inventory (Oldfield, 1971) and reported using English as a first language. Knowledge of vocabulary was assessed using the National Adult Reading Test (NART) (Nelson and Wilson, 1991). The Mini-Mental State Examination (MMSE) (Folstein et al., 1975) was also administered to the older group as a test to confirm the absence of cognitive impairment. None of the participants from either group reported a history of neurological disease, head trauma, alcoholism, mental illness, or cerebral tumor and all had sufficient vision and hearing to perform the task. Hearing thresholds were also confirmed in the older group using a pure tone audiometry test to rule out significant hearing impairment and all average thresholds were under 40 dB (*M* = 28.55, *SD* = 3.41). Ethical approval for this study was received from the University of Queensland Medical Research Ethics Committee and the Queensland Health Human Research Ethics Committee. Site-specific research governance was also obtained from the Royal Brisbane and Women's Hospital Ethics Committee.

### Procedure

The lexical decision task was explained to each participant prior to the scanning session and a computer-based practice task administered until a score of over 80% was achieved. The fMRI task consisted of 120 individual stimuli, presented binaurally via MRI confon headphones (MR Confon GmbH, Magdeburg, Germany) across two runs within the same scanning session (refer to Figure 1 in Roxbury et al., 2014 for acquisition sequence). At the start of each trial participants saw a small black fixation cross (48 point font) for 2.3 s and a visual prompt “Is it a real word?” A word or pseudoword was then heard by the participant with a mean length of auditory presentation lasting an average of 764 ms (*SD* = 105 ms). Participants had 3.5 s to make a response and during this response/sound window, a screen with a black “+” and the correct orientation for Yes/No was displayed. Participants selected their response using an MR compatible button press box (Current Designs Inc., Philadelphia PA). A left button press signaled a positive (yes) response and a right button press signaled a negative (no) response. A large blue cross (84 point font) appeared on the screen to indicate that a response had been selected and remained visible for 1 s. After this time, a black cross (84 point font) appeared and stayed on screen until the start of the next trial. Participants were asked to keep their eyes open for the duration of the scanning session and to look at the fixation cross. Participants used their left hand to make their responses on the button response box in order to allow for a future comparison study on patients with aphasia following stroke.

Stimuli were presented in a pseudorandomised, event related design with a maximum of two consecutive trials from any one condition (concrete real words, abstract real words and pseudowords). To reduce order effects, five different pseudorandomised orders were employed. In addition, words and pseudowords which shared constituent syllables were not presented in consecutive order. An inter-trial interval (jittered between 10 and 18 s, mean 14 s) followed each auditory stimulus presentation. The long interval was designed to allow for future comparison of this data with that of patients with post-stroke aphasia, who can experience a delay in hemodynamic response function (Bonakdarpour et al., 2007).

### Data acquisition

Participants underwent one scanning session at the Royal Brisbane and Women's Hospital with a Siemens 3 Tesla Trio scanner (Siemens Erlangen). A pseudorandomised event-related design was employed and stimuli delivered in two runs lasting ~14.4 min in duration. During the two task runs, a total of 390 gradient echo EPI images with BOLD sensitivity were acquired (TR 2210 ms; TE 30 ms; slice thickness 3 mm with 0.3 mm gap; 36 axial slices, FOV 220 × 220 mm, flip angle 90, matrix 64 × 64). At the start of the scanning session, a 3D T1 weighted image was also acquired [MP-RAGE; TR 1900 ms; TE 2.4 ms; TI 900 ms; (0.9 mm)^3^ resolution].

### Image processing

Statistical parametric mapping software (SPM8; Wellcome Trust Centre for Neuroimaging; http://www.fil.ion.ucl.ac.uk/spm) was used to process and analyze the images with MATLAB 2009a (The MathWorks Inc., Natick, MA). The first five volumes acquired at the beginning of each run were discarded to ensure that images were only included for analysis once magnetization had reached steady state. EPI images from run 1 and run 2 were realigned using INRIAlign (Freire et al., 2002) to correct for motion artifacts and a mean image created. This mean EPI was then coregistered with the T_1_ image acquired in the same session and the T_1_ image segmented and spatially normalized (Ashburner and Friston, 2005). A DARTEL template (Ashburner, 2007) was created for both groups and each subject's T_1_ and EPI image normalized to the standard Montreal Neurological Institute (MNI) space. The EPI images were then resampled (3.0 × 3.0 × 3.0 mm^3^) and spatially smoothed using an 8 mm full-width half-maximum (FWHM) Gaussian smoothing kernel.

### Behavioral analysis

Mean accuracy was calculated per subject. Reaction times were calculated from the beginning of the sound onset and all incorrect trials plus those that were shorter than 100 ms were removed prior to analysis. Both mean accuracy and reaction time data were not normally distributed (Shapiro-Wilk test of normality: accuracy *p* < 0.003; RT *p* < 0.0001). As group variances could be treated as equal (using a non-parametric Levene's test) a Mann–Whitney test was employed. In addition, to determine whether the concreteness effect existed for each group we compared concrete and abstract processing differences associated with accuracy and reaction time, using a Wilcoxon Signed-Ranks test.

### Imaging data analysis

A fixed effects analysis was employed for each subject. The general linear model was constructed using a hemodynamic response function with derivatives. This was done to model the increased variability due to aging (D'Esposito et al., 2003). The realignment parameters (6° of freedom) were included as regressors of no interest. In order to exclude BOLD response effects which were due to variability in reaction time (or time on task), a parametric modulation for each of the three conditions (concrete, abstract and pseudoword) was included using the mean corrected reaction time for each trial. Contrasts included concrete—abstract, concrete—pseudoword and abstract—pseudoword. Error trials were modeled separately, and included both incorrect and trials where the reaction time was <100 ms.

For the whole brain analyses, a group by condition (2 × 3) factorial analysis was completed to determine the main effects. Anatomy Toolbox (Eickhoff et al., 2007) was used to determine the neuroanatomical locations of peak maxima for significant clusters elicited in the different contrasts. To correct for multiple comparisons, a Monte Carlo simulation calculation was performed and cluster thresholds calculated by using the FWHM of the square root of the residuals (3dFWHMx and 3dClustSim; Analysis of Functional Neuroimages; Cox, 1996). Using a height threshold of *p* < 0.001 uncorrected, a family wise error rate of *p* < 0.05 was achieved with a minimum cluster threshold of 44 contiguous voxels. Regions showing a main effect of condition or group were investigated further to explore directionality and mean percent signal change calculated for each region.

A priori regions of interest (ROIs), determined from Binder et al. (2009) and Cabeza et al. (2002), were created using IBASPM 116 Human Atlas in WFU PickAtlas (Maldjian et al., 2003, 2004) in SPM8 (Wellcome Trust Centre for Neuroimaging; http://www.fil.ion.ucl.ac.uk/spm). A total of 10 ROIs were created; six to examine concrete word processing (left AG, right AG, left MFG, left SFG, left posterior cingulate, and left fusiform), three for abstract processing, left IFG; pars orbitalis and left aSTS—split into anterior superior temporal gyrus (aSTG) and anterior middle temporal gyrus (aMTG). An additional ROI in right IFG (pars orbitalis) was included, to investigate recruitment of homologous, contralateral brain regions particularly the ventral, inferior frontal cortex by older adults (Cabeza et al., 2002). The anterior, superior and middle temporal cortical regions were subdivided according to the delineation of *y* < −7 for anterior by Indefrey and Levelt (2004) so that potential activity in the aSTS could be thoroughly examined.

Mean percent signal for each participant in each of the ROIs was extracted using MarsBaR (Brett et al., 2002), a region of interest toolbox for SPM8. SPSS Statistics version 21 (IMB; Armonk, New York, USA) was used for group statistical analyses. Data was checked for normality of distribution and when not normally distributed a Log_10_ transformation was applied to create normality. Two (young, old) × 3 (concrete, abstract, and pseudoword) repeated measures ANOVAs were then conducted to test for mean percent signal change differences between conditions within the 10 ROIs. Where the assumption of sphericity had been violated, degrees of freedom were corrected using Huynh-Feldt estimates of sphericity and are reported throughout along with original degrees of freedom. *Post-hoc* pairwise comparisons (concrete—abstract, concrete—pseudoword, abstract—pseudoword) in the 10 ROIs were evaluated using an adjusted p level following correction for multiple comparisons (Benjamini and Hochberg, 1995). Where results indicated a group by condition interaction, an ANOVA was conducted to explore the interactions within a single group between conditions.

## Results

### Participant results

There was no significant difference between cohorts on either the NART score [*p* = 0.2, young mean 32.18 (SD 5.07), older mean 35.18 (SD 7.95)] or mean years of education [*p* = 0.5, young mean 16.5 yrs (SD 2.1), older mean 15.5 yrs (SD 4)]. Scores from the MMSE (Folstein et al., 1975) confirmed intact cognition of the older group (max = 30, group mean = 28.82, *SD* = 0.95).

### Behavioral results

The mean accuracy for all conditions was high in both groups. Mann–Whitney *U*-tests between the groups indicated that accuracy of pseudowords was significantly greater for young adults (*M* = 97.9, *SD* = 2.3) than for older adults (*M* = 95.2, *SD* = 4.1) (*U* = 79.5, *Z* = −2.295, *p* = 0.022), but there was no significant difference between the groups for accuracy in either the concrete (young *M* = 98.6, *SD* = 2.4; older *M* = 97.1, *SD* = 4.4) or abstract conditions (young *M* = 97.5, *SD* = 1.9; older *M* = 93.5, *SD* = 7.5).

The percentage of reaction times removed (incorrect or <100 ms) was 4.6% for older adults and 2% for younger adults. A Mann–Whitney *U*-test on mean response times for the young and old groups (see Table 1 below) indicated that young adults were significantly faster than older adults for all three conditions; pseudoword (*U* = 311,864, *Z* = −13.746, *p* < 0.001 two-tailed), concrete (*U* = 100,275, *Z* = −5.319, *p* < 0.001 two-tailed), and abstract (*U* = 97,875, *Z* = −4.807, *p* < 0.001 two-tailed).

**Table 1:** **Mean reaction times for young and older adults**.

	**Pseudoword**	**Concrete**	**Abstract**
	**Mean (SD)**	**Mean (SD)**	**Mean (SD)**
Young adults	1434 ms (396)	1187 ms (274)	1263 ms (346)
Older adults	1738 ms (588)	1288 ms (318)	1356 ms (353)

Concreteness effects, or the processing advantage seen for concrete over abstract words in terms of accuracy and reaction time, were tested in both the young and old groups. The older group was significantly more accurate for concrete over abstract words (*Z* = −2.116, *p* = 0.034) but there was no significant difference for the younger group (*p* = 0.303). The results for reaction times indicate faster processing for concrete over abstract words, with a significant difference for both the young group (*Z* = −15.316, *p* < 0.001) and the older group (*Z* = −13.241, *p* < 0.001) (see Table 1) and confirm that the expected processing advantage for concrete words occurred.

### Region of interest analyses

Results for the left fusiform ROI indicated that there was a main effect of group *F*_(1, 16)_ = 7.523, *p* = 0.014, with older adults eliciting increased activity in this region (*M* = 0.304, *SE* = 0.062) compared to young adults (*M* = 0.207, *SE* = 0.041). A main effect of group was also observed in the left AG *F*_(1, 16)_ = 38.329, *p* = 0.001, with older adults eliciting increased activity (*M* = 0.282, *SE* = 0.028) compared to young adults (*M* = −0.065, *SE* = 0.035). No other region showed a main effect of group.

A main effect for condition was found for left AG, right AG, left posterior cingulate, left aMTG, left MFG, left SFG, left aSTG, and left IFG. Regions showing significant main effects for condition effects after FDR correction (Benjamini and Hochberg, 1995) are listed in Table 2.

**Table 2:** **Region of interest—main effect of condition**.

**Region**	***F*-value**	**Concrete—Pseudoword**	**Abstract—Pseudoword**	**Concrete—Abstract**
Left AG	*F*_(2, 15)_ = 9.643,	Conc > PS	Ab > PS	Conc > Ab
	*p* = 0.002	*p* < 0.001	*p* = 0.017	*p* = 0.017
Right AG	*F*_(2, 15)_ = 13.315,	Conc > PS	Ab > PS	Conc > Ab
	*p* < 0.001	*p* < 0.001	*p* = 0.009	*p* = 0.030
Left posterior cingulate	*F*_(2, 15)_ = 16.161,	Conc > PS	Ab > PS	Conc > Ab
	*p* < 0.001	*p* < 0.001	*p* = 0.013	*p* = 0.002
Left aMTG	*F*_(2, 15)_ = 8.101,	Conc > PS	Ab > PS	*ns*
	*p* = 0.004	*p* = 0.009	*p* = 0.002	
Left MFG	*F*_(2, 15)_ = 6.04,	Conc > PS	Ab > PS	*ns*
	*p* = 0.012	*p* = 0.009	*p* = 0.03	
Left SFG	*F*_(2, 15)_ = 11.260,	Conc > PS	Ab > PS	*ns*
	*p* = 0.001	*p* < 0.001	*p* = 0.017	
Left aSTG	*F*_(2, 15)_ = 18.568,	Conc > PS	PS > Ab	*ns*
	*p* < 0.001	*p* < 0.001	*p* = 0.03	
Left IFG	*F*_(2, 15)_ = 10.124,	PS > Conc	*ns*	Ab > Conc
	*p* = 0.002	*p* = 0.047		*p* < 0.001

**Figure 1:**
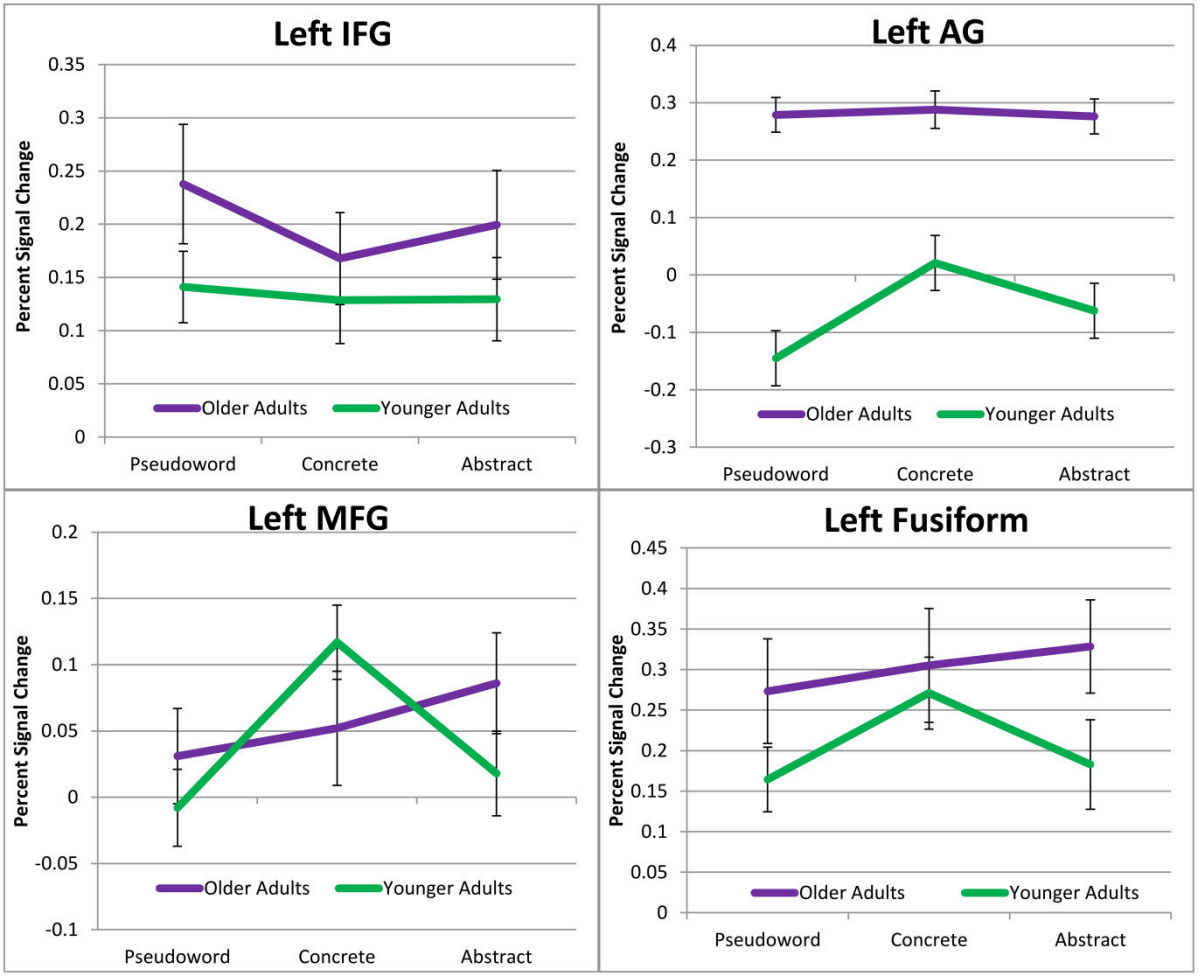
**Region of interest analysis**. Illustrates ROIs which showed a significant group by condition interaction.

Results from the repeated measures ANOVA for each of the 10 ROIs revealed a significant age × condition interaction in four ROIs; left AG *F*_(1.431, 22.903)_ = 9.102, *p* = 0.003 (Huynh-Feldt correction), left IFG *F*_(2, 15)_ = 8.936, *p* = 0.003, left fusiform *F*_(2, 15)_ = 6.531, *p* = 0.009, and left MFG *F*_(2, 15)_ = 6.040, *p* = 0.011. Follow-up analyses on the significant interactions were conducted to find the differences in the mean percent change between conditions within each group. The interactions and mean percent BOLD signal change in the four ROIs can be seen in Figure 1. There was a condition effect in the left IFG for the older adults only *F*_(2, 15)_ = 17.127, *p* = 0.001. *Post-hoc* pairwise comparisons indicated significant differences for the concrete compared to both the pseudoword (*p* = 0.001) and abstract (*p* < 0.001) conditions. Mean percent signal change was greater for the abstract (*M* = 0.20, *SE* = 0.05) and pseudowords (*M* = 0.24, *SE* = 0.06) compared to the concrete words (*M* = 0.17, *SE* = 0.04). Results also indicated a significant difference in activity for condition in the left AG for the young adults only *F*_(2, 15)_ = 11.270, *p* = 0.001. Pairwise comparisons indicated that the differences between contrasts were significant for all three conditions (*p* ≤ 0.001) with greatest activity for the concrete condition (*M* = 0.02, *SE* = 0.04), followed by abstract words (*M* = −0.06, *SE* = 0.04) and then pseudowords (*M* = −0.14, *SE* = 0.04).

An effect of condition was observed in the left MFG for both the older adults *F*_(2, 15)_ = 4.268, *p* = 0.034 and young adults *F*_(2, 15)_ = 9.897, *p* = 0.002. *Post-hoc* pairwise comparisons further indicated a significant difference in the older adults for the abstract—pseudoword contrast (*p* = 0.018) with mean percent signal change elicited being significantly higher for abstract (*M* = 0.09, *SE* = 0.04) compared to pseudowords (*M* = 0.03, *SE* = 0.04). A significant difference was observed in the young group for concrete words when compared to both abstract (*p* = 0.003) and pseudoword conditions (*p* < 0.001). Mean percent signal change indicated that greatest activity was elicited for the concrete words (*M* = 0.12, *SE* = 0.03) compared to abstract (*M* = 0.02, *SE* = 0.03) and pseudoword (*M* = −0.01, *SE* = 0.03) conditions. A condition effect was also observed in the left fusiform for both groups [older adults *F*_(2, 15)_ = 10.114, *p* = 0.002 and young adults *F*_(2, 15)_ = 14.952, *p* < 0.001]. Pairwise comparisons between conditions indicated significant differences in the older adults for abstract and pseudoword condition (*p* = 0.001) with abstract words eliciting greater activity (*M* = 0.33, *SE* = 0.06) than pseudowords (*M* = 0.27, *SE* = 0.07). Significant differences for the young group were observed for the concrete—pseudoword (*p* < 0.001) and abstract—pseudoword condition (*p* = 0.044). Mean percent signal change was greater for both the concrete (*M* = 0.27, *SE* = 0.04) and abstract conditions (*M* = 0.18, *SE* = 0.06) when compared to pseudowords (*M* = 0.16, *SE* = 0.04).

### Whole brain analyses

We also ran exploratory whole brain analyses which revealed a main effect of age in the left precentral, left supplementary motor area (SMA), left IFG (pars triangularis), and right IFG (pars triangularis extending into pars opercularis) for the word greater than pseudoword contrast. A main effect of condition was also observed in the left calcarine gyrus (refer Table 3). There were no significant group by condition interactions for the whole-brain analyses.

**Table 3:** **Factorial results from whole brain analysis for main effects between young and older groups**.

**Main effect**	**Structure**	***x***	***y***	***z***	**Volume**	***z*-score**
Age	Left precentral gyrus	−36	4	32	53	4.65
	Right IFG (pars triangularis)	54	18	22	65	4.62
	Left SMA	−7	25	47	62	4.62
	Left IFG (pars triangularis)	−50	32	18	138	4.45
Condition	Left calcarine gyrus	−7	−50	7	76	4.54

p < 0.001 probability threshold and cluster threshold level of 44 voxels.

**Figure 2:**
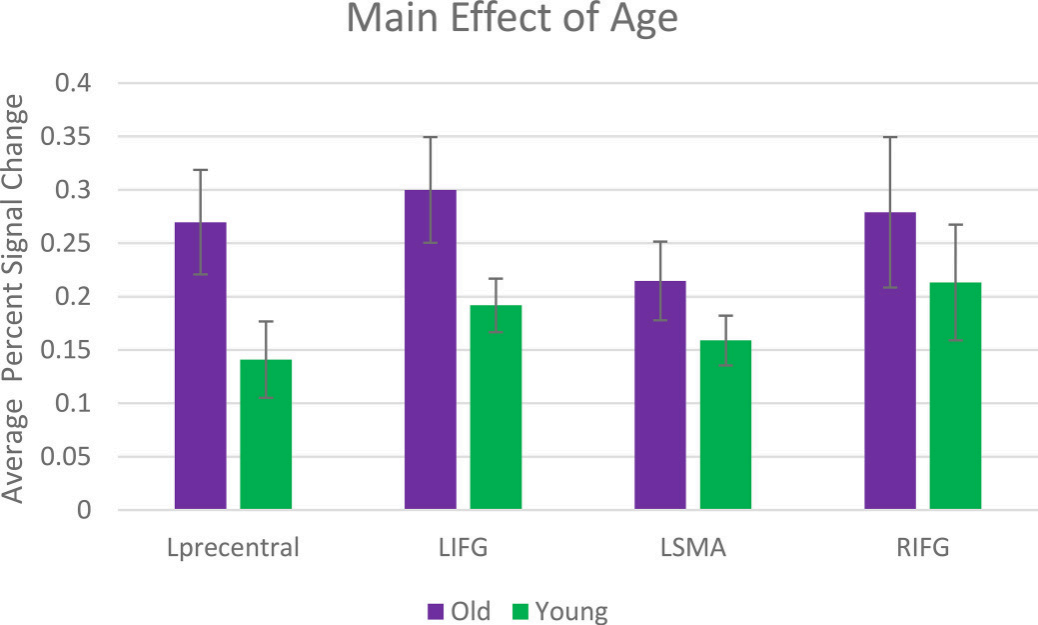
**Main effect of age**. Activation differences were calculated using the mean percent signal change extracted for each cluster.

Brain regions in which a main effect of age was indicated were investigated further to explore directionality of the effect. Differences in activity were calculated using the mean percent signal change extracted for each cluster (see Figure 2) and show that older adults elicited increased activity in all four regions compared to the young adults.

Mean percent signal change was also calculated to explore the main effect of condition in the calcarine gyrus. Results indicate that concrete words elicited increased activity (*M* = 0.06, *SD* = 0.04) in this region than abstract words (*M* = 0.07, *SD* = 0.04) across groups.

## Discussion

We investigated concreteness effects during spoken word comprehension in young vs. old adults and tested the competing age-related hypotheses of attenuation (Peters and Daum, 2008) vs. preservation (Tyler et al., 2010; see Wingfield and Grossman, 2006 for a review) in concrete and abstract word processing. We also examined whether behavioral performance was associated with age-related changes in neural activity. We found that comprehension of spoken concrete and abstract words and the concreteness effect is indeed preserved in aging. However, the neural substrates that underpin this preserved performance appear to vary as a function of age and may reflect compensatory age-related upregulation.

The expected processing advantage for concrete over abstract words was observed in both groups for reaction time and for accuracy in the older adults only. The lack of concreteness effect in accuracy for the young cohort was most likely due to a ceiling effect. Results for accuracy between the two groups for concrete and abstract words revealed no significant differences. These findings are consistent with previously reported observations in the healthy aging literature, which suggests that spoken word comprehension remains relatively preserved throughout life (Burke and Shafto, 2008; Shafto et al., 2012). A significant difference in accuracy did emerge for the pseudoword condition and showed that older adults were less accurate than the young adults when responding to these non-lexical items. With regard to reaction times, our results showed that the older adults were significantly slower than their younger counterparts for all three conditions. This result is in agreement with previous observations of a consistent, general slowing in lexical decision response times as people age (Madden, 1992; Gold et al., 2009). Whilst both groups responded to pseudowords slower than both concrete and abstract words, the response time observed for the older adults was dramatically slower compared to the young adults. This is consistent with the model of Balota and Chumbley (1984) which proposes that fast lexical decision responses can be facilitated by the familiarity or unfamiliarity of the stimuli. Since, the pseudowords employed in this study were opaque (Raettig and Kotz, 2008), and aging is associated with an increase in lexical knowledge (Burke and Shafto, 2008), the slower response time observed for the older adults to the pseudowords might be due to a more extensive lexical search which is required before a decision can be made.

In the left IFG ROI, older adults showed increased activity for abstract and pseudowords compared to concrete words, whereas young adults showed no difference between conditions. This recruitment by the older adults of a mechanism not used by the young group for abstract and pseudoword processing, accompanied by a preserved performance suggests an element of age-related compensatory upregulation. Studies investigating differences between semantic and phonological processes and IFG activity have shown that different linguistic processing components can be differentially affected in aging (Meinzer et al., 2009, 2012; Geva et al., 2012; Shafto et al., 2012; Diaz et al., 2014). Previous studies investigating concreteness have shown involvement of left inferior frontal regions when abstract words are directly contrasted with concrete words (Mellet et al., 1998; Perani et al., 1999; Wise et al., 2000; Fiebach and Friederici, 2004; Noppeney and Price, 2004; Binder et al., 2005) and this may represent strategic retrieval of semantic knowledge (Fliessbach et al., 2006). However, the fact that this region was also activated more for pseudowords compared to concrete words, but not when directly contrasted with abstract words makes it unlikely that upregulation of this region by the older adults reflects semantic-based processes. Rather, we suggest that the current findings are more consistent with previous studies which have indicated a role for left IFG in more phonologically-based, short term working memory processes (Fiebach and Friederici, 2004; Binder et al., 2005; Sabsevitz et al., 2005). This is supported by results from a study investigating differences in word learning strategies in aging, which demonstrated that when older adults are engaged in word learning tasks, they rely more on phonological processes, and less on semantic working memory (Service and Craik, 1993). Our findings are also in agreement with reports that pseudoword processing activates more focal phonological processes compared to real words which activate lexical representations at a higher level (Davis and Gaskell, 2009). Thus, since we observed greater activation for abstract and pseudowords compared to concrete words in left IFG for the older adults only, we propose the involvement of the left IFG may instead reflect an age-related compensatory upregulation of more phonologically-based, rather than semantically-mediated or working memory processes.

Young adults showed increased left AG activity for concrete words compared to abstract words, with both concrete and abstract words also showing greater activity than pseudowords. In contrast, older adults showed no change in AG activity for the different conditions. Involvement of the left AG in semantic-based processing generally is well-documented (Price, 2010) and this was also the region identified in the Binder et al. (2009) meta-analysis as being most reliably activated in response to semantic-based tasks. Findings from our previous study investigating concreteness effects in healthy young adults showed that the left AG was the region most robustly activated by concrete compared to abstract words (Roxbury et al., 2014). Recent work has suggested that the AG may act as a supramodal zone, binding and integrating sensory-motor information from modality-specific regions (Binder and Desai, 2011). The findings in the present study are consistent with this view and demonstrate that the young adults are reliably using this region when accessing semantic conceptual knowledge. However, this is not the case for the older adults. While older adults do recruit left AG, and generally elicit greater activity than the young adults, no activation differences between the conditions suggests that older adults are recruiting the left AG for more general lexical processing. As such, we propose that this reduction in specificity may be due to a change in focus by the older adults who attend more to phonological rather than semantic aspects of processing in order to maintain a preserved performance.

In the present study, we observed increased activation for abstract compared to pseudowords in left MFG for the older adults. The MFG has generally been considered to be associated with more executive type functions relating to working memory, inhibition and processing speed (Grady, 2008; Reuter-Lorenz and Park, 2010) and thus the finding of increased activation for abstract words by the older adults might reflect an engagement of executive resources. This increased neural activity for the older adults for abstract word processing is supported by the behavioral findings which showed both slower reaction times and reduced accuracy for the abstract condition suggesting that abstract words required more effortful processing. However, we also observed increased activation in this region for the young group for the concrete words compared to both abstract and pseudowords and this was associated with an intact behavioral performance both in terms of reaction time and accuracy. These findings are not therefore consistent with an executive function explanation for this region. Instead, activity in this region has previously been observed in semantic-based tasks although its precise function in language processing is not yet well understood. Thus, the finding of increased activity for concrete words in the young adults could potentially reflect processes involved with the retrieval of semantic knowledge (Binder et al., 2009; Peelle et al., 2010; Diaz et al., 2014), which is greater for concrete concepts due to their richer set of conceptual features.

In the left fusiform gyrus older adults showed a generalized increase in activity overall and elicited greater activity for abstract words compared to pseudowords. In contrast, the young adults reliably recruited this region for the processing of real words (concrete and abstract) compared to pseudowords. The left fusiform gyrus is considered to be a conceptual semantic store and associated with the retrieval of visual attributes (Binder et al., 2009). Since concrete words are associated with stronger visual attributes than abstract words, the expectation is that concrete words will elicit increased activity in this region. However, while some have shown increased activity in this region for concrete compared to abstract words (D'Esposito et al., 1997; Mellet et al., 1998; Wise et al., 2000; Fiebach and Friederici, 2004; Whatmough et al., 2004), others have not (Kiehl et al., 1999; Perani et al., 1999; Friederici et al., 2000; Jessen et al., 2000; Noppeney and Price, 2004; Binder et al., 2005). The findings in this present study differ from those of Whatmough et al. (2004) who observed increased activity for concrete words in the left fusiform gyrus and these differences may be due to the nature of the lexical decision task we employed with does not require deeper conceptual processing. Nevertheless, our finding of increased activity in the young group for both concrete and abstract words compared to the pseudowords suggests that lexical processing was occurring and we tentatively interpret this result as reflecting differences associated with real word imagery and features, compared to pseudowords that have no meaning and therefore no imagery or features attached to them. Meanwhile, the increased activity for the abstract compared to pseudoword condition for the older group might suggest that the older adults were engaged in increased visual imagery for retrieving abstract words in order to maintain a preserved performance.

Results for the whole brain analysis revealed a main effect of age in left IFG (pars triangularis, extending into pars opercularis), right IFG (pars triangularis), left precentral gyrus, and left supplementary motor area (SMA) with older adults eliciting increased activity in each of these regions compared to the young adults for words compared to pseudowords. The increase in bilateral PFC activity is consistent with the HAROLD model (Cabeza, 2002) which proposes that when performing the same task, older adults elicit additional activity in the contralateral right PFC regions (Cabeza et al., 2004). However, the HAROLD model also predicts that activity elicited by older adults will be less-lateralized than that observed in young adults. The current results do not support a reduction in laterality in the older group since we also observed increased activity in the left hemisphere in the older compared to young adults. This trend toward a general increase in brain activity in the older compared to the young adults suggests a compensatory upregulation of brain regions, which are required to maintain performance in word processing.

The fMRI data in the present study confirm involvement of a large network of common regions which are activated in response to both concrete and abstract words regardless of age. Of the eight regions which showed condition effects, three ROIs (left AG, right AG, and left posterior cingulate) showed greater activation for concrete greater than abstract greater than pseudowords. The results for increased activation for concrete compared to abstract words in left and right AG are consistent with Binder and Desai's (2011) view of embodied abstraction which proposes that bilateral AG acts as a convergence zone with the purpose of binding conceptual representations from modal-specific regions. Differences in processing in this region are associated with the different amounts of conceptual information associated with a word. As such, in this framework, concrete words would be expected to elicit more activity than abstract words due to their stronger semantic representations, largely associated with sensory-motor knowledge (Binder and Desai, 2011). With regard to the two prominent theories of concreteness and abstract processing, the results for bilateral AG activity cannot be fully explained by either account. Context availability theory does not predict increased right hemisphere activity for abstract or concrete concepts, since quantitative differences between these word types should occur in left-hemisphere regions (Schwanenflugel and Shoben, 1983). In terms of dual coding theory, involvement of the nonverbal right hemisphere is predicted by the model for concrete words, due to their richer image-based associations. However, we also observed a significant condition effect in the left IFG with greater activity for abstract compared to concrete words. While this is consistent with dual coding theory, which predicts that abstract words will activate qualitatively distinct systems in the verbal left-hemisphere, we also observed greater activity for pseudowords compared to concrete words in this region which is not predicted by the model. Instead, as discussed above, this finding suggests that the older adults recruit left IFG as a compensatory mechanism in order to maintain performance during more phonologically-based processes.

In summary, our results show that, despite a general reduction in response times, spoken language comprehension of concrete and abstract words, and concreteness effects remain relatively preserved in aging. Interestingly, the pseudoword condition proved most problematic for the healthy older adults and we suggest that may be due to the additional strategic processing required as they search through a more extensive lexicon (Kemper and Sumner, 2001; Verhaeghen, 2003) before deciding to discard an item. The results from the imaging data showed that a large network of brain regions, previously reported as being involved in concrete and abstract processing (Binder et al., 2009; Wang et al., 2010), are similarly activated by both groups in response to concrete and abstract words although the older adults routinely showed increased activation compared to the young adults. Age-related vascular changes need to be considered when comparing activity between young and older adults (D'Esposito et al., 2003). A general reduction in BOLD has been observed previously, meaning a decrease in activation cannot be directly tied to a decrease in neural activity. However, this doesn't explain the increased activity in older compared to younger adults seen in this study, which is contrary to the expected direction based on possible biological differences, and indicates a larger neuronal activation or BOLD response. Selective regions also showed activation differences between conditions for the two groups. The findings for left IFG and left AG present an interesting dichotomy. These findings suggest that while the spoken recognition of concrete and abstract processing remains preserved in aging, this preserved performance is accompanied by compensatory upregulation in regions which are differentially recruited by the two groups such that older adults are required to focus more on phonological and less on semantic aspects of processing and this appears essential for preservation of functionality.

## Author contributions

DC, KM, and TR conceived the study and contributed to the study design, analysis and interpretation of the imaging data. TR developed the stimuli and was responsible for the acquisition of data. AC provided analysis of neuroimaging data. TR wrote the paper and DC, KM and AC provided guidance on the manuscript drafts, approved the final version and agreed to be accountable for the work.

### Conflict of interest statement

The authors declare that the research was conducted in the absence of any commercial or financial relationships that could be construed as a potential conflict of interest.
